# The Shift From Efficacy to Implementation Science (2020-2026) in Nursing Practice for Digital Mental Health: Scoping Review and Bibliometric Analysis

**DOI:** 10.2196/91498

**Published:** 2026-07-08

**Authors:** Shoukai Yu, Lingmei Qian, Hao Wang

**Affiliations:** 1 School of Medicine, Shanghai Jiao Tong University, 720 Xianxia Road, Room 104, Shanghai, Shanghai, 20000, China, 86 18917625425; 2 Hongqiao International Institute of Medicine, Shanghai Tongren Hospital and Clinical Research Institute, Shanghai Jiao Tong University School of Medicine, Shanghai, Shanghai, China; 3 Tongren Hospital, Shanghai Jiao Tong University School of Medicine, Shanghai, Shanghai, China

**Keywords:** digital mental health, mobile health, mHealth, eHealth, implementation science, nursing informatics, bibliometric analysis, scoping review, workflow integration, mobile phone

## Abstract

**Background:**

Nurses are pivotal as end users and implementers of digital mental health interventions (DMHIs). However, the successful translation of efficacious DMHIs into sustainable nursing practice is hindered by multifaceted implementation challenges.

**Objective:**

This study aimed to systematically map and analyze the evolving research landscape of nurse-involved DMHIs to determine if a paradigm shift from efficacy testing to implementation science is occurring and characterize the methodological and thematic trends associated with this shift.

**Methods:**

We conducted a scoping review integrated with a bibliometric analysis following the PRISMA-ScR (Preferred Reporting Items for Systematic Reviews and Meta-Analyses extension for Scoping Reviews) guidelines. A comprehensive search of the Web of Science Core Collection, Scopus, and PubMed databases (January 2020 to April 2026) yielded 1014 eligible primary research studies. Studies were classified as “efficacy/effectiveness” or “implementation” research using a validated framework. We analyzed temporal trends and methodological designs and performed co-word thematic mapping.

**Results:**

Implementation research constituted the largest proportion of clearly classified studies (327/470, 69.6%), exceeding efficacy research (143/470, 30.4%). Implementation research demonstrated a significant growth trajectory from 2020 to 2025 (β=8.63 articles per year; *P*=.003), surpassing efficacy research in 2022. Implementation studies used more mixed methods (105/327, 32.1% vs 7/143, 4.9%; *P*<.001) and qualitative designs (92/327, 28.1% vs 2/143, 1.4%; *P*<.001). Thematic analysis revealed “nursing workflow integration” and “nurse-led implementation” as emerging motor themes.

**Conclusions:**

This review provides robust evidence of an accelerating turn to implementation in digital mental health research in nursing. The field is moving beyond internal validity toward real-world implementation. Future efforts should focus on implementation-ready digital tools and embedding implementation science competencies into nursing education.

## Introduction

The integration of digital mental health interventions (DMHIs) into routine nursing practice represents both an opportunity and a challenge for contemporary health care systems [1-3]. Nurses, as the largest group of health care professionals worldwide, are uniquely positioned to serve as frontline implementers, coordinators, and sustainers of DMHIs across diverse clinical settings [4,5]. However, despite growing evidence of DMHI efficacy from over a decade of randomized controlled trials [6,7], their real-world implementation in nursing practice remains limited, creating what has been termed the “implementation-to-practice gap” in digital mental health [8,9].

Recent systematic reviews have consistently identified nurse-specific barriers to DMHI adoption, including workflow disruption, inadequate training, and concerns about increased workload [10,11]. A 2020 mixed methods study by Bourla et al [12] found that although over 70% of student nurses reported willingness to adopt digital mental health technologies in clinical practice, more than 60% voiced worries that such tools could damage the therapeutic relationship, indicating that the readiness of future nursing practitioners to implement DMHIs remains insufficient. Concurrently, the global nursing shortage, exacerbated by the COVID-19 pandemic, has intensified interest in digital solutions that can extend the reach of mental health services [13,14].

Implementation science offers conceptual frameworks and methodological approaches specifically designed to address these implementation challenges [15,16]. Frameworks such as the reach, effectiveness, adoption, implementation, and maintenance framework [17] and the Consolidated Framework for Implementation Research [18] shift the research focus from questions of efficacy (“Does it work under ideal conditions?”) to questions of implementation (“How can nurses successfully integrate this into their workflow?”). While calls for greater attention to implementation science in nursing digital health research have increased [19,20], the extent to which the published literature reflects this methodological paradigm shift remains empirically unexamined.

To inform the development of more implementable digital tools and effective support strategies for nurses, this scoping review and bibliometric analysis systematically mapped the published literature from 2020 to 2026. We sought to determine whether nurse-involved DMHI research is indeed undergoing this critical implementation turn. Our specific objectives were to (1) quantify the prevalence of implementation-focused research vs efficacy research, (2) analyze temporal trends in this paradigm adoption, (3) compare the methodological approaches characterizing each paradigm, and (4) identify the key thematic foci and their evolution within the nursing DMHI literature. A conservative keyword-based classification strategy was used to prioritize specificity, providing a lower-bound estimate of implementation research prevalence.

## Methods

### Study Design

This study was conducted as a scoping review incorporating a bibliometric analysis in accordance with the PRISMA-ScR (Preferred Reporting Items for Systematic Reviews and Meta-Analyses extension for Scoping Reviews) statement [21]. The review protocol was not preregistered. Our primary objective was to systematically map and characterize the published literature on nurse-involved DMHIs from 2020 to 2026. The completed PRISMA-ScR checklist is provided in Checklist 1.

### Data Source and Search Strategy

Data were retrieved from 3 electronic databases on April 15, 2026: Web of Science Core Collection, Scopus, and PubMed. The search was limited to peer-reviewed journal articles published in English between January 1, 2020, and April 15, 2026. The search strategy combined terms related to three conceptual domains: (1) digital interventions [22,23], (2) mental health conditions [3], and (3) nursing involvement and research paradigms [4,8]. The complete search strategy for each database is provided in Multimedia Appendix 1.

### Inclusion and Exclusion Criteria

Articles were included if they met all of the following criteria: (1) focused primarily on a digital intervention targeting mental health conditions; (2) involved nursing roles (nurse-led, nurse-delivered, or nurse-involved implementation); (3) reported original empirical research with human participants; (4) were published in English in peer-reviewed journals; and (5) were published between January 1, 2020, and April 15, 2026. We excluded review articles, study protocols, commentaries, editorials, theoretical papers, and studies in which nursing involvement was peripheral.

### Article Classification and Validation

Publications were classified using a multistage process informed by implementation science frameworks [18,24]. Implementation research was defined by a focus on real-world adoption and implementation outcomes in nursing contexts [25], and efficacy and effectiveness research was defined by a focus on clinical outcomes under controlled conditions. A conservative keyword-based classification strategy was used to prioritize specificity, providing a lower-bound estimate of implementation research prevalence. Classification followed 3 stages: automated keyword screening, manual verification, and validation against a consensus standard (κ=0.73, 95% CI 0.60-0.86).

### Data Extraction and Quality Assessment

Two reviewers independently extracted data from each included study using a standardized form. Extracted items included first author, publication year, country, study design, sample size, research paradigm (implementation vs efficacy or effectiveness), theoretical framework used, implementation outcomes measured (if applicable), and key themes. Disagreements were resolved through discussion or consultation with a third reviewer. Quality assessment was not performed as this was a scoping review focused on mapping the literature rather than critically appraising evidence quality [21].

### Statistical and Bibliometric Analysis

Descriptive statistics summarized publication characteristics. Proportions with 95% CIs were calculated using the Wilson score method. Temporal trends were analyzed using linear regression. Methodological differences between research paradigms were examined using chi-square tests. Co-word analysis and thematic mapping were conducted using the *bibliometrix* package in R (version 4.2; R Foundation for Statistical Computing) [26].

### Ethical Considerations

This study involved analysis of publicly available published literature and did not require ethics approval.

## Results

### Study Selection and Characteristics

**Figure 1.**
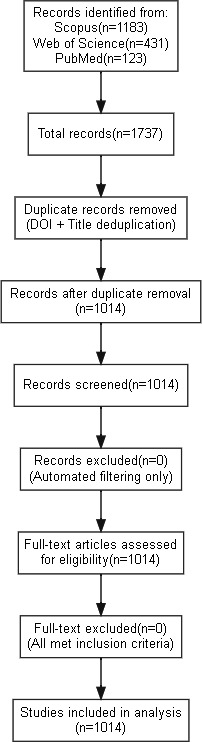
PRISMA (Preferred Reporting Items for Systematic Reviews and Meta-Analyses) flowchart. DOI: digital object identifier.

Our search identified 1737 records across 3 databases (Scopus: n=1183, 68.1%; Web of Science: n=431, 24.8%; PubMed: n=123, 7.1%). To ensure a conservative estimate of unique publications, we performed deduplication using both digital object identifier (DOI) and title. Of the 1737 identified records, DOI-based deduplication removed 337 (19.4%) duplicates (resulting in n=1400, 80.6% of records), and subsequent title-based deduplication identified an additional 386 (22.2%) duplicate records with inconsistent or missing DOI assignments across databases. This 2-stage approach yielded 1014 unique records (Figure 1). All 1014 records met the inclusion criteria for original empirical research on nurse-involved DMHIs published in English between 2020 and 2026.

The final sample included studies from 44 countries. The largest contributions came from the United States (373/1014, 36.8%), the United Kingdom (161/1014, 15.9%), Australia (110/1014, 10.8%), and China (83/1014, 8.2%). The mean sample size of primary studies was 162 (SD 241) participants.

Across all 1014 included studies, randomized controlled trials represented the most common study design (n=303, 29.9%). Observational designs followed as the second most frequent category (n=129, 12.7%), followed by mixed methods research (n=106, 10.5%). Feasibility and pilot studies and qualitative studies each comprised 7.1% (n=72) of the studies, whereas process evaluation designs were the least prevalent (n=44, 4.3%).

### Prevalence of Research Paradigms

For research paradigm classification, a conservative keyword coding approach left 53.6% (544/1014) of the total studies with an ambiguous categorization that could not be clearly assigned to either implementation- or efficacy-focused work. Among the remaining 470 clearly classified studies, implementation-focused research constituted the dominant paradigm at 69.6% (n=327), whereas efficacy and effectiveness research made up the remaining 30.4% (n=143). All mixed or hybrid methodological studies were subsumed within the implementation research grouping in line with standard implementation science classification frameworks [27]. The implementation-to-efficacy study ratio was calculated at 2.29:1, demonstrating a preponderance of implementation research within the clearly classified sample.

### Temporal Trends in Paradigm Adoption

**Figure 2.**
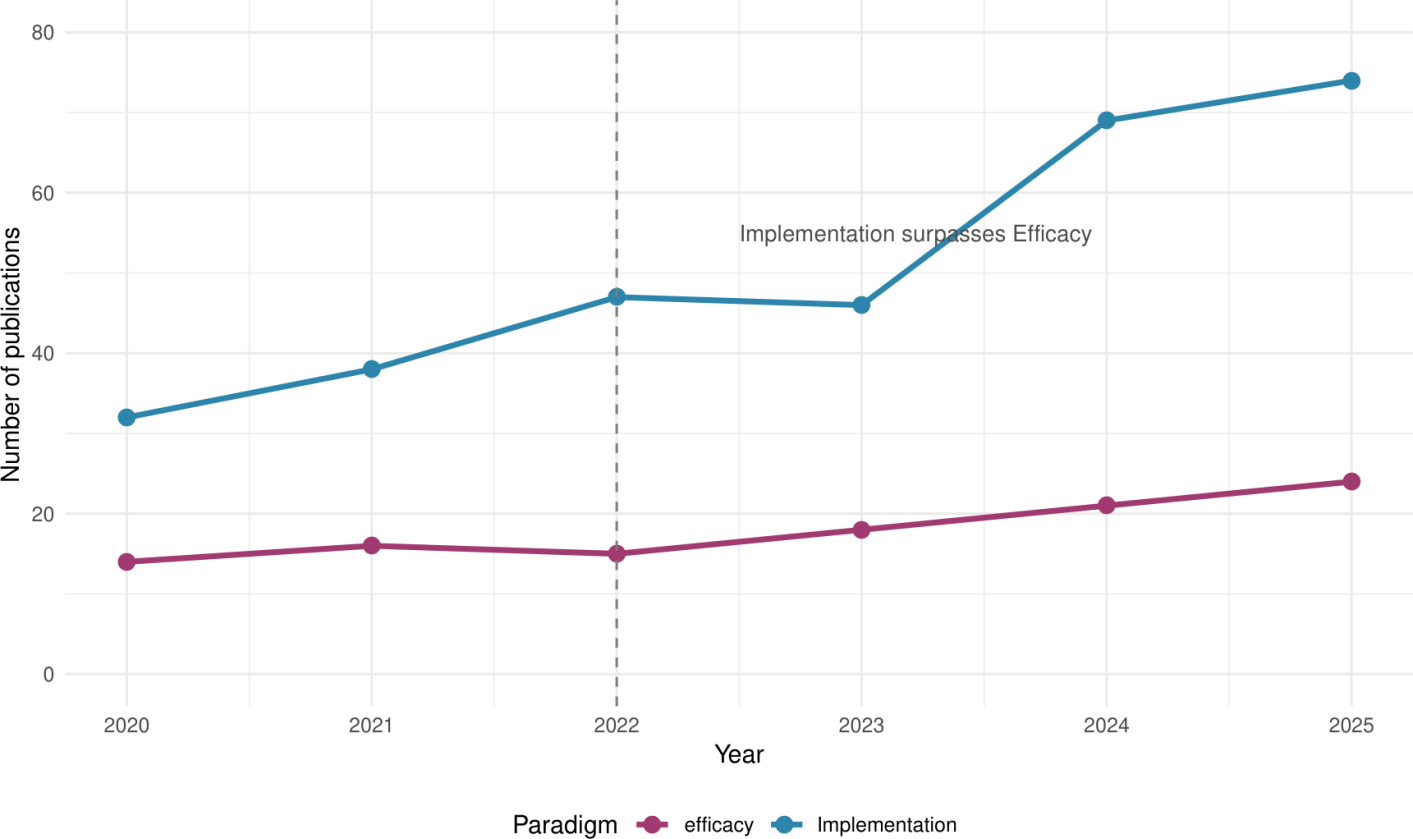
Annual publication trends by research paradigm (2020-2025). Implementation research demonstrated a significant growth trajectory, increasing from 32 articles in 2020 to 74 in 2025, surpassing efficacy research in 2022 (47 vs 15). Linear regression analysis (2020-2025) revealed a β value of 8.63 articles per year (95% CI 5.21-12.05; *P*=.003; *R*^2^=0.91). Efficacy and effectiveness research showed no significant growth (β=1.8; *P*=.48). The year 2026 was excluded due to incomplete data.

Publication volume demonstrated substantial growth from 2020 to 2025 (Figure 2), increasing from 102 articles in 2020 to 247 in 2025 (142% increase). Linear regression analysis revealed a significant positive trend in implementation research from 2020 to 2025 (β=8.63 articles per year, 95% CI 5.21-12.05; *P*=.003; *R*^2^=0.91). Implementation research studies outnumbered efficacy research studies in 2022 (47 implementation-related articles vs 15 efficacy or effectiveness articles) and retained this predominance in subsequent years, peaking at 74 articles in 2025. Efficacy and effectiveness research demonstrated no statistically significant growth across the study period (β=1.8 articles per year; *P*=.48). The lower count in 2026 (21 implementation articles) reflects incomplete year data and was excluded from trend analysis.

### Methodological Divergence Between Paradigms

**Figure 3.**
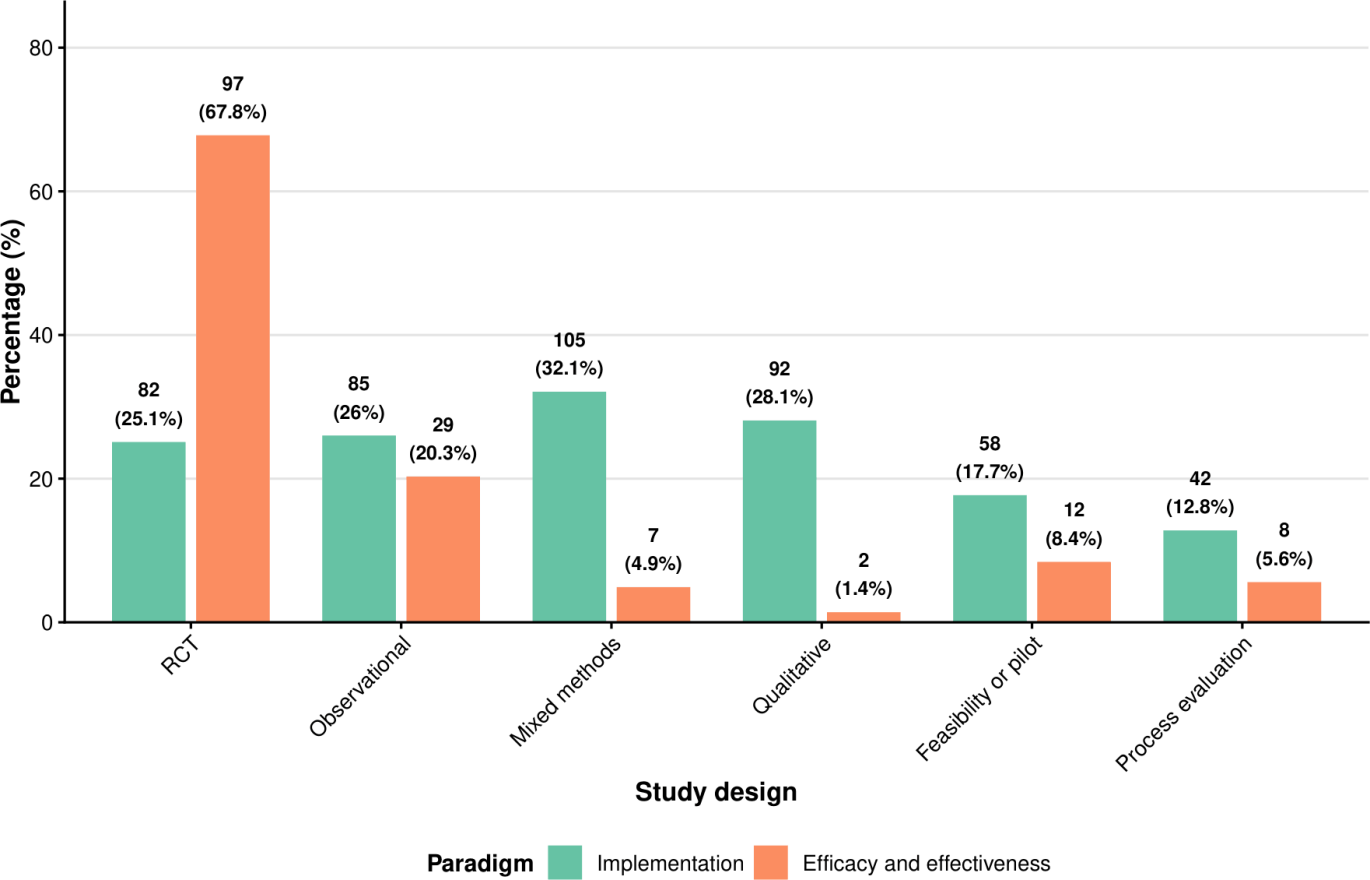
Methodological approaches across research paradigms (n=470 studies with unambiguous classification). Implementation research used significantly more mixed methods (105/327, 32.1% vs 7/143, 4.9%; *P*<.001) and qualitative designs (92/327, 28.1% vs 2/143, 1.4%; *P*<.001) compared to efficacy and effectiveness research, which remained predominantly reliant on randomized controlled trials compared to implementation studies (97/143, 67.8% vs 82/327, 25.1%, respectively; *P*<.001). RCT: randomized controlled trial.

Analysis of studies with unambiguous classification revealed substantial methodological divergence (Table 1 and Figure 3). Implementation studies used significantly more mixed methods designs (105/327, 32.1% vs 7/143, 4.9%; *χ*^2^_5_=32.4; *P*<.001) and qualitative approaches (92/327, 28.1% vs 2/143, 1.4%; *χ*^2^_5_=41.2; *P*<.001) compared to efficacy and effectiveness studies. In contrast, efficacy research remained predominantly reliant on randomized controlled trials compared to implementation studies (97/143, 67.8% vs 82/327, 25.1%, respectively; *χ*^2^_5_=24.8; *P*<.001).

**Table 1.** Methodological approaches across research paradigms (n=470 studies with unambiguous classification).^a^

Study design	Implementation research (n=327), n (%)	Efficacy and effectiveness research (n=143), n (%)	Chi-square (*df*)	*P* value
RCT^b^	82 (25.1)	97 (67.8)	24.8 (5)	<.001
Observational	85 (26.0)	29 (20.3)	1.8 (5)	.41
Mixed methods	105 (32.1)	7 (4.9)	32.4 (5)	<.001
Qualitative	92 (28.1)	2 (1.4)	41.2 (5)	<.001
Feasibility or pilot	58 (17.7)	12 (8.4)	2.3 (5)	.32
Process evaluation	42 (12.8)	8 (5.6)	3.9 (5)	.14

a Percentages exceed 100% because studies may use multiple designs. Chi-square tests compare implementation vs efficacy and effectiveness research.

b RCT: randomized controlled trial.

### Thematic Evolution and Nursing-Specific Patterns

**Figure 4.**
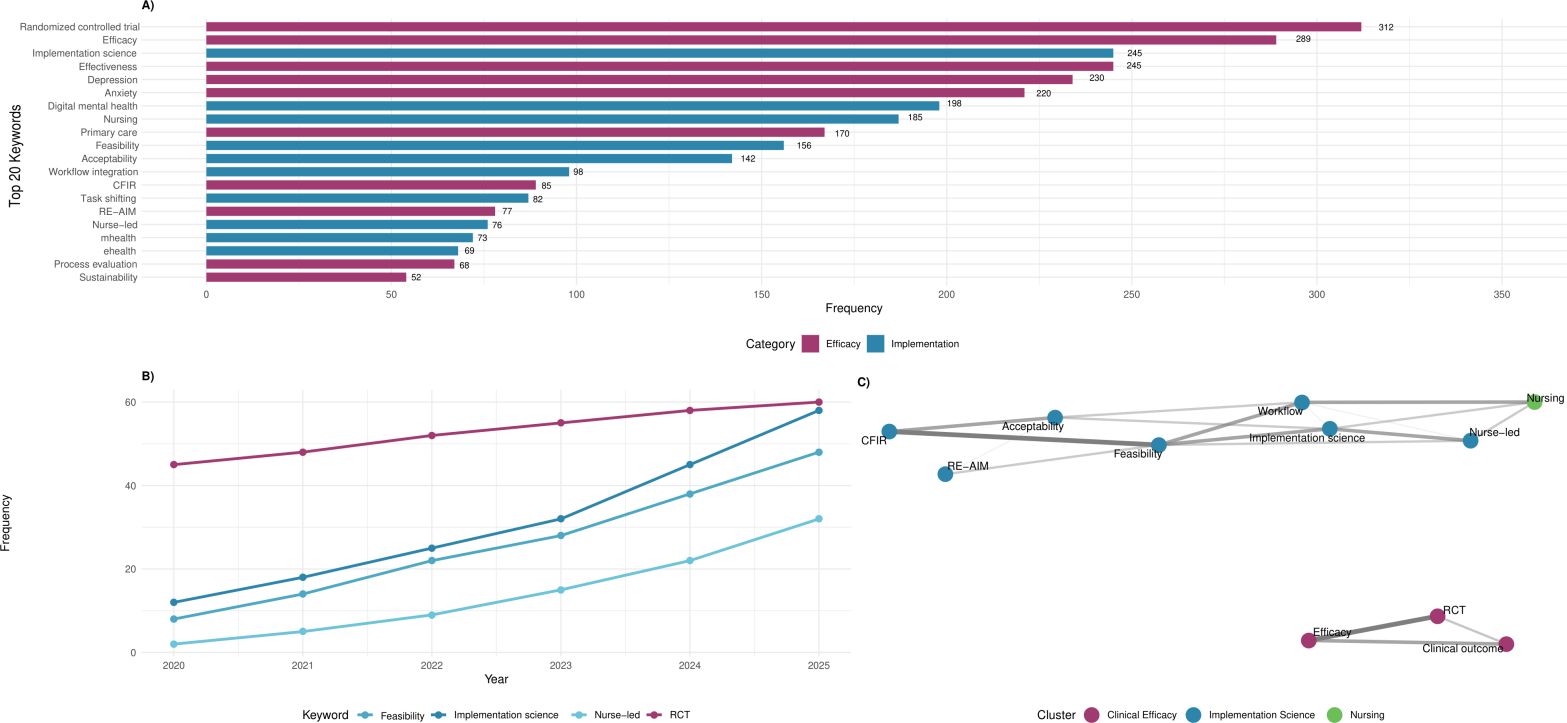
Thematic evolution in nurse-involved digital mental health research (N=1014). (A) Bar chart of the top 20 most frequently occurring keywords across all the included studies. Implementation-related keywords (blue) dominate the top ranks, with “randomized controlled trial” (n=312) and “efficacy” (n=289) being the most frequent efficacy-related terms. (B) Annual trends of the top 10 keywords (2020-2025), demonstrating increasing prominence of implementation-related terms (“implementation science,” “feasibility,” and “nurse-led”), whereas efficacy-related terms (“RCT”) remained stable. The year 2026 was excluded due to incomplete data. (C) Keyword co-occurrence network showing three distinct clusters: (1) implementation science (blue; centrality=0.85), (2) clinical efficacy research (purple; centrality=0.32), and (3) nursing workflow integration (green; centrality=0.68). Implementation science emerged as a motor theme with high centrality and density.

Co-word analysis of keywords from all 1014 publications revealed a clear thematic evolution toward implementation science (Figure 4). Implementation-focused constructs including “implementation science,” “nursing workflow,” “task-shifting,” and “nurse-led implementation” emerged as motor themes characterized by high centrality and density. Nursing-specific implementation barriers such as “time constraints,” “training needs,” and “workflow integration” showed increasing prominence in recent publications (2024‐2025). Notably, implementation-related keywords showed the highest annual growth rate among all thematic categories (β=2.1 appearances per year; *P*=.008).

## Discussion

### Principal Findings

Our bibliometric analysis of 1014 nurse-involved DMHI studies provides robust empirical evidence of a decisive paradigm shift toward implementation science. Three convergent lines of evidence support this conclusion. First, implementation research constituted the largest proportion of clearly classified studies (327/470, 69.6%), exceeding traditional efficacy research (143/470, 30.4%). Second, implementation research demonstrated a significant annual growth rate of 8.63 articles per year from 2020 to 2025 (*P*=.003; *R*^2^=0.91), surpassing efficacy research in 2022. Third, fundamental methodological differences characterize these paradigms, with implementation studies using substantially more mixed methods (105/327, 32.1% vs 7/143, 4.9%) and qualitative designs (92/327, 28.1% vs 2/143, 1.4%).

### Comparison With Existing Literature

Our findings align with broader trends in implementation science but extend them to the specific context of nursing digital health [28,29]. Previous research has documented increasing attention to implementation science in health care generally [30], but our study is the first to quantify this shift specifically in nurse-involved DMHI research using a multi-database bibliometric approach. The methodological divergence we observed—with implementation studies favoring mixed methods approaches—reflects the complex, context-dependent nature of implementation challenges in nursing practice [27].

Recent studies have begun to identify effective nurse-specific implementation strategies. Targeted, blended training combining online learning and in-person coaching markedly elevates nurses’ adoption of digital mental health tools, with intervention groups demonstrating more than double the uptake relative to untrained control staff [31]. Similarly, research confirms that “implementation champions” drive long-term sustainability; grassroots nurse-led digital interventions maintain stable clinical use far longer than institution-mandated top-down rollouts [32].

### Implications for Nursing Practice

The documented shift has direct implications for creating more nurse-friendly digital health ecosystems.

First, for digital health developers, our findings underscore the necessity of “implementation by design.” DMHI development must move beyond user experience for patients to include clinician experience for nurses, explicitly considering workflow integration, time burden, and interoperability with clinical systems from the earliest stages [22,23].

Second, for nursing informatics leadership, chief nursing informatics officers and other leaders must advocate for and allocate resources toward implementation infrastructure. This includes dedicated time for nurse training and adaptation, the creation of role-specific implementation playbooks, and the evaluation of DMHIs based on implementation outcomes (eg, adoption, feasibility, and sustainability) alongside clinical outcomes [33].

Third, for education and training, nursing curricula and continuing professional development must evolve to build digital health implementation competency. This includes skills in evaluating digital tools for implementability, leading practice change, and using data from DMHIs to inform care—a core component of modern nursing informatics literacy [34,35].

### Future Directions for Nursing Research

Our findings suggest several directions for future nursing research. First, there is a need for more hybrid effectiveness-implementation designs that simultaneously examine clinical outcomes and implementation processes [27]. Second, research should explore nurse-specific implementation strategies such as tailored training programs and workflow integration tools [36]. Third, future studies should examine how implementation outcomes vary across nursing roles and settings [37]. Finally, given the geographic concentration of research in high-income countries, there is an urgent need for implementation research in low- and middle-income settings, where nurses often serve as the primary mental health care providers [38].

### Strengths and Limitations

Strengths of this study include its comprehensive multi-database search strategy (Web of Science, Scopus, and PubMed), rigorous classification system with validation (κ=0.73), updated search up to April 2026, and integration of multiple bibliometric techniques, analyzing 1014 eligible studies.

Several limitations warrant acknowledgment. First, reliance on title and abstract text for initial classification may have misclassified some studies, although manual verification and consensus validation mitigated this risk. Our conservative classification strategy prioritized specificity, resulting in a higher proportion of unclear classifications (544/1014, 53.6%); this provides a lower-bound estimate of implementation research prevalence. Second, the exclusion of non–English-language publications introduces language bias, potentially missing relevant research from non–English-speaking countries. Third, we did not include CINAHL due to institutional access limitations, which may have underrepresented nursing-specific literature. Future reviews should include this database. Fourth, the rapid evolution of digital health technologies means that findings from older studies (2020-2022) may not fully reflect current capabilities, although our temporal trend analysis addresses changing patterns over time. Fifth, the lack of quality assessment, consistent with scoping review methodology, means that we cannot comment on the methodological rigor of the included studies.

### Conclusions

This scoping review and bibliometric analysis documents a decisive and accelerating paradigm shift toward implementation science in nurse-involved DMHI research. Implementation research now constitutes the dominant paradigm (327/1014, 32.2% vs 143/1014, 14.1% for efficacy research) and demonstrated a significant annual growth rate of 8.63 articles per year from 2020 to 2025 (*P*=.003; *R*^2^=0.91), surpassing efficacy research in 2022. The field is expanding beyond a primary focus on internal validity to systematically investigate the complex determinants and processes of real-world implementation in nursing contexts.

## Supplementary material

10.2196/91498 Multimedia Appendix 1 Complete search strategy.

10.2196/91498 Checklist 1 PRISMA-ScR checklist.

## Abbreviations

DMHI: digital mental health intervention
DOI: digital object identifier
PRISMA-ScR: Preferred Reporting Items for Systematic Reviews and Meta-Analyses

## References

[1] Naslund JA, Bondre A, Torous J, Aschbrenner KA (2020). Social media and mental health: benefits, risks, and opportunities for research and practice. J Technol Behav Sci.

[2] Torous J, Andersson G, Bertagnoli A (2019). Towards a consensus around standards for smartphone apps and digital mental health. World Psychiatry.

[3] Lattie EG, Stiles-Shields C, Graham AK (2022). An overview of and recommendations for more accessible digital mental health services. Nat Rev Psychol.

[4] Janes G, Chesterton L, Heaslip V (2025). Current nursing and midwifery contribution to leading digital health policy and practice: an integrative review. J Adv Nurs.

[5] Booth RG, Strudwick G, McBride S, O’Connor S, Solano López AL (2021). How the nursing profession should adapt for a digital future. BMJ.

[6] Andrews G, Basu A, Cuijpers P (2018). Computer therapy for the anxiety and depression disorders is effective, acceptable and practical health care: an updated meta-analysis. J Anxiety Disord.

[7] Weisel KK, Fuhrmann LM, Berking M, Baumeister H, Cuijpers P, Ebert DD (2019). Standalone smartphone apps for mental health-a systematic review and meta-analysis. NPJ Digit Med.

[8] Graham AK, Lattie EG, Powell BJ (2020). Implementation strategies for digital mental health interventions in health care settings. Am Psychol.

[9] Granja C, Janssen W, Johansen MA (2018). Factors determining the success and failure of eHealth interventions: systematic review of the literature. J Med Internet Res.

[10] Borghouts J, Eikey E, Mark G (2021). Barriers to and facilitators of user engagement with digital mental health interventions: systematic review. J Med Internet Res.

[11] Li Y, Ji W, Chen H, Xie X, Yang J, Gao J (2024). Psychometric properties of instruments used to measure the informatics competence of nurses: a systematic review. Nurse Educ Pract.

[12] Bourla A, Mouchabac S, Ogorzelec L, Guinchard C, Ferreri F (2020). Are student nurses ready for new technologies in mental health? Mixed-methods study. Nurse Educ Today.

[13] (2020). State of the world’s nursing 2020: investing in education, jobs and leadership. World Health Organization.

[14] Drissi N, Ouhbi S, Marques G, de la Torre Díez I, Ghogho M, Janati Idrissi MA (2021). A systematic literature review on e-mental health solutions to assist health care workers during COVID-19. Telemed J E Health.

[15] Bauer MS, Damschroder L, Hagedorn H, Smith J, Kilbourne AM (2015). An introduction to implementation science for the non-specialist. BMC Psychol.

[16] Nilsen P (2015). Making sense of implementation theories, models and frameworks. Implement Sci.

[17] Glasgow RE, Vogt TM, Boles SM (1999). Evaluating the public health impact of health promotion interventions: the RE-AIM framework. Am J Public Health.

[18] Damschroder LJ, Aron DC, Keith RE, Kirsh SR, Alexander JA, Lowery JC (2009). Fostering implementation of health services research findings into practice: a consolidated framework for advancing implementation science. Implement Sci.

[19] Al-Moteri M, Aljuaid J (2025). Development of an implementation science higher diploma for registered nurses: phase III of the EQUIP initiative. Nurs Health Sci.

[20] Fontaine G, Vinette B, Weight C (2024). Effects of implementation strategies on nursing practice and patient outcomes: a comprehensive systematic review and meta-analysis. Implement Sci.

[21] Tricco AC, Lillie E, Zarin W (2018). PRISMA extension for scoping reviews (PRISMA-ScR): checklist and explanation. Ann Intern Med.

[22] Greenhalgh T, Wherton J, Papoutsi C (2017). Beyond adoption: a new framework for theorizing and evaluating nonadoption, abandonment, and challenges to the scale-up, spread, and sustainability of health and care technologies. J Med Internet Res.

[23] Mair FS, May C, O’Donnell C, Finch T, Sullivan F, Murray E (2012). Factors that promote or inhibit the implementation of e-health systems: an explanatory systematic review. Bull World Health Organ.

[24] Kirk MA, Kelley C, Yankey N, Birken SA, Abadie B, Damschroder L (2016). A systematic review of the use of the Consolidated Framework for Implementation Research. Implement Sci.

[25] Proctor E, Silmere H, Raghavan R (2011). Outcomes for implementation research: conceptual distinctions, measurement challenges, and research agenda. Adm Policy Ment Health.

[26] Aria M, Cuccurullo C (2017). bibliometrix: an R-tool for comprehensive science mapping analysis. J Informetr.

[27] Curran GM, Bauer M, Mittman B, Pyne JM, Stetler C (2012). Effectiveness-implementation hybrid designs: combining elements of clinical effectiveness and implementation research to enhance public health impact. Med Care.

[28] Jha R, Sachdeva G (2026). Digital mapping of health-related quality of life research in non-communicable diseases: a topic modeling and bibliometric synthesis for policy and practice. Inform Health Soc Care.

[29] Falagas ME, Pitsouni EI, Malietzis GA, Pappas G (2008). Comparison of PubMed, Scopus, Web of Science, and Google Scholar: strengths and weaknesses. FASEB J.

[30] Palinkas LA, Aarons GA, Horwitz S, Chamberlain P, Hurlburt M, Landsverk J (2011). Mixed method designs in implementation research. Adm Policy Ment Health.

[31] Boß L, Ross J, Reis D (2025). Effectiveness of an integrated platform-based intervention for promoting psychosocial safety climate and mental health in nursing staff: a pragmatic cluster randomised controlled trial. Int J Nurs Stud.

[32] Borges do Nascimento IJ, Abdulazeem H, Vasanthan LT (2023). Barriers and facilitators to utilizing digital health technologies by healthcare professionals. NPJ Digit Med.

[33] Laakkonen N, Jarva E, Hammarén M (2024). Digital competence among healthcare leaders: a mixed-methods systematic review. J Nurs Manag.

[34] Kleib M, Nagle LM, Furlong KE, Paul P, Duarte Wisnesky U, Ali S (2022). Are future nurses ready for digital health?: informatics competency baseline assessment. Nurse Educ.

[35] Belchez CA, Kunkel D, Kulhanek B, Tietze MF, Pinekenstein B (2026). Integrating digital health and informatics competencies in nursing curricula: a framework for nurse educators. Nurse Educ.

[36] Powell BJ, Waltz TJ, Chinman MJ (2015). A refined compilation of implementation strategies: results from the Expert Recommendations for Implementing Change (ERIC) project. Implement Sci.

[37] Lynch EA, Mudge A, Knowles S, Kitson AL, Hunter SC, Harvey G (2018). “There is nothing so practical as a good theory”: a pragmatic guide for selecting theoretical approaches for implementation projects. BMC Health Serv Res.

[38] Ibeneme S, Karamagi H, Muneene D, Goswami K, Chisaka N, Okeibunor J (2022). Strengthening health systems using innovative digital health technologies in Africa. Front Digit Health.

