# Supplementary material for: The Shift From Efficacy to Implementation Science (2020-2026) in Nursing Practice for Digital Mental Health: Scoping Review and Bibliometric Analysis
Source: JMIR Nurs. 2026 Jul 8;9:e91498. doi: 10.2196/91498 (PMC13344321; doi:10.2196/91498)
Supplement: Multimedia Appendix 1 [file nursing-v9-e91498-s001.docx]

**Multimedia Appendix 2: Complete Search Strategy**

**Complete Search Strategies for Web of Science Core Collection, Scopus, and PubMed Databases**

This appendix provides the complete search strategy used for each database, including search terms, Boolean operators, filters, and retrieval dates, corresponding to the scoping review and bibliometric analysis titled “Digital Mental Health in Nursing Practice: A Scoping Review and Bibliometric Analysis of the Shift from Efficacy to Implementation Science (2020-2026)”. All searches were conducted on April 15, 2026, covering the period from January 1, 2020, to April 15, 2026.

**1. Web of Science Core Collection Search Strategy**

**Search Terms (Boolean Combination):**

((“digital mental health” OR “mHealth” OR “eHealth” OR “digital intervention” OR “mobile health” OR “telehealth” OR “telemedicine” OR “digital therapy”) AND (“mental health” OR “psychiatric” OR “depression” OR “anxiety” OR “schizophrenia” OR “bipolar disorder” OR “mental illness”) AND (“nurse*” OR “nursing” OR “nursing practice” OR “implementation science” OR “implementation research” OR “efficacy research” OR “effectiveness research”))

**Filters Applied:**

- Document Type: Article (peer-reviewed)
- Language: English
- Publication Year: 2020–2026
- Indexes: SCI-EXPANDED, CCR-EXPANDED, IC

**Retrieval Results:** 431 records

**2. Scopus Search Strategy**

**Search Terms (Boolean Combination):**

((“digital mental health” OR “mHealth” OR “eHealth” OR “digital intervention” OR “mobile health” OR “telehealth” OR “telemedicine” OR “digital therapy”) AND (“mental health” OR “psychiatric” OR “depression” OR “anxiety” OR “schizophrenia” OR “bipolar disorder” OR “mental illness”) AND (“nurse*” OR “nursing” OR “nursing practice” OR “implementation science” OR “implementation research” OR “efficacy research” OR “effectiveness research”))

**Filters Applied:**

- Document Type: Article
- Language: English
- Publication Year: 2020–2026
- Source Type: Peer-reviewed Journals

**Retrieval Results:** 1,183 records

**3. PubMed Search Strategy**

**Search Terms (Boolean Combination):**

((“digital mental health”[MeSH Terms] OR “mHealth”[MeSH Terms] OR “eHealth”[MeSH Terms] OR “digital intervention”[Title/Abstract] OR “mobile health”[Title/Abstract] OR “telehealth”[MeSH Terms] OR “telemedicine”[MeSH Terms] OR “digital therapy”[Title/Abstract]) AND (“mental health”[MeSH Terms] OR “psychiatric disorders”[MeSH Terms] OR “depression”[MeSH Terms] OR “anxiety disorders”[MeSH Terms] OR “schizophrenia”[MeSH Terms] OR “bipolar disorder”[MeSH Terms] OR “mental illness”[Title/Abstract]) AND (“nurses”[MeSH Terms] OR “nursing”[MeSH Terms] OR “nursing practice”[Title/Abstract] OR “implementation science”[MeSH Terms] OR “implementation research”[Title/Abstract] OR “efficacy research”[Title/Abstract] OR “effectiveness research”[Title/Abstract]))

**Filters Applied:**

- Publication Date: 2020/01/01 to 2026/04/15
- Language: English
- Article Type: Journal Article
- Humans: Yes

**Retrieval Results:** 123 records

**Note:** After retrieval, duplicate records were removed using both DOI and title matching, resulting in 1,014 unique eligible studies included in the final analysis (see Supplementary Figure S1 for the PRISMA flow diagram).
