# Supplementary material for: The Shift From Efficacy to Implementation Science (2020-2026) in Nursing Practice for Digital Mental Health: Scoping Review and Bibliometric Analysis
Source: JMIR Nurs. 2026 Jul 8;9:e91498. doi: 10.2196/91498 (PMC13344321; doi:10.2196/91498)
Supplement: Checklist 1 [file nursing-v9-e91498-s002.docx]

**Multimedia Appendix 1: PRISMA-ScR Checklist**

**Preferred Reporting Items for Systematic Reviews and Meta-Analyses Extension for Scoping Reviews (PRISMA-ScR) Checklist**

This checklist is completed in accordance with the PRISMA-ScR statement [21], corresponding to the scoping review and bibliometric analysis titled “Digital Mental Health in Nursing Practice: A Scoping Review and Bibliometric Analysis of the Shift from Efficacy to Implementation Science (2020-2026)”. All items are marked as “Yes” (Y), “No” (N), or “Not Applicable” (NA), with additional explanations where necessary.

| PRISMA-ScR Item | Y/N/NA | Explanation |
| --- | --- | --- |
| Title: Include a title that clearly indicates the study is a scoping review | Y | Title explicitly states “A Scoping Review and Bibliometric Analysis” |
| Abstract: Provide a structured abstract (background, objectives, methods, results, conclusions) | Y | Abstract follows the structured format, covering all required sections |
| Introduction: Provide context and rationale for the scoping review | Y | Introduction elaborates on the significance of DMHIs in nursing and the research gap |
| Objectives: Specify the research objectives and research questions | Y | Four specific objectives are clearly stated in the Introduction section |
| Methods: Specify the study design (scoping review) | Y | Study design is clearly defined as a scoping review integrated with bibliometric analysis |
| Methods: Specify the eligibility criteria for included studies | Y | Inclusion and exclusion criteria are detailed in the Methods section |
| Methods: Describe the information sources and search strategy | Y | Three databases (Web of Science, Scopus, PubMed) and search details are provided; complete strategy in Appendix 2 |
| Methods: Describe the process of study selection | Y | Study selection process (search, deduplication, inclusion/exclusion) is detailed; PRISMA flow diagram in Supplementary Figure S1 |
| Methods: Describe the data extraction process | Y | Data extraction by two independent reviewers, with third reviewer resolving disagreements |
| Methods: Describe the data items extracted | Y | Extracted items include author, year, country, study design, sample size, research paradigm, etc. |
| Methods: Describe how data were analyzed and synthesized | Y | Descriptive statistics, linear regression, chi-square tests, and co-word analysis (bibliometrix package in R) are used |
| Results: Present the results of the search and study selection | Y | Search results (1,737 records, 1,014 eligible) and selection process are detailed |
| Results: Present the characteristics of included studies | Y | Characteristics (geographic distribution, sample size, study design) are presented in Results |
| Results: Present the results of data synthesis | Y | Synthesis includes paradigm prevalence, temporal trends, methodological divergence, and thematic evolution |
| Discussion: Interpret the results in the context of the objectives and existing literature | Y | Discussion links results to research objectives and compares with existing literature |
| Discussion: Address limitations of the scoping review | Y | Limitations (classification bias, language bias, CINAHL exclusion, etc.) are detailed |
| Discussion: Provide implications for practice, policy, or research | Y | Implications for nursing practice, research, education are presented in Discussion |
| Funding: Disclose funding sources | Y | Funding from Shanghai Pujiang Talent Program (19PJC085) is disclosed |
| Conflict of Interest: Disclose conflicts of interest | Y | No conflicts of interest are declared |
